# Understanding the Complexities of Cast Post Retention: A Comprehensive Review of Influential Factors

**DOI:** 10.7759/cureus.51258

**Published:** 2023-12-28

**Authors:** Neha K Urkande, Nikhil Mankar, Pradnya P Nikhade, Manoj Chandak

**Affiliations:** 1 Conservative Dentistry and Endodontics, Sharad Pawar Dental College and Hospital, Datta Meghe Institute of Higher Education & Research, Wardha, IND

**Keywords:** biomechanics, cad/cam technology, digital impressions, dental materials, restorative dentistry, cast post retention

## Abstract

This comprehensive review delves into the intricate landscape of cast post retention in restorative dentistry, encompassing historical perspectives, contemporary techniques, and future directions. Examining factors ranging from tooth-related considerations to prosthesis-related dynamics, the review provides a detailed analysis of clinical techniques, including step-by-step procedures, common challenges, and innovative advancements. Technological breakthroughs, such as digital impressions, computer-aided design and computer-aided manufacturing (CAD/CAM) technology, three-dimensional (3D) printing, and finite element analysis, are explored for their transformative impact on precision and customization. The discussion extends to the promising future of cast post retention, emphasising emerging materials, the integration of artificial intelligence in treatment planning, and patient-specific approaches. Implications for clinical practice underscore the importance of individualised treatment planning and the adoption of advanced technologies. Recommendations for future research advocate for comprehensive long-term clinical studies, investigations into AI-driven treatment planning, and a focus on patient outcomes and satisfaction. This review consolidates existing knowledge and anticipates a future marked by enhanced precision, individualised care, and improved long-term success in cast post-retained restorations.

## Introduction and background

Dentistry has witnessed significant advancements in restorative techniques, and one area that continues to garner attention is cast post-retention. Cast posts reinforce endodontically treated teeth, providing stability and support for subsequent restorations. This comprehensive review aims to delve into the intricate nuances of cast post retention, exploring its definition, purpose, and pivotal role in restorative dentistry [1].

Cast post retention refers to utilizing custom-fabricated metal posts, typically cast in alloys like base metal, to enhance the structural integrity of teeth following root canal treatment. Incorporating cast posts into restorative procedures also provides a foundation for base metal. By anchoring securely within the root canal space, these posts contribute to the overall stability of the restoration, ensuring its longevity and functionality [2].

The importance of cast post retention in restorative dentistry is underscored by the inherent challenges of endodontically treated teeth. These teeth often face compromises in structural integrity due to the absence of vital pulp tissue, rendering them susceptible to fractures. Cast posts are pivotal in addressing this vulnerability by acting as internal reinforcements. These posts contribute to the even distribution of occlusal forces through their strategic placement, thereby minimizing the risk of structural failure. This enhances the restoration's overall longevity and preserves the natural tooth structure, a fundamental consideration within the broader context of conservative dentistry [3].

This review seeks to comprehensively examine the multifaceted aspects influencing cast post retention. Each facet will be scrutinised from historical perspectives to contemporary clinical techniques to offer a nuanced understanding of the complexities involved. By exploring the various factors impacting the success of cast post-retained restorations, clinicians and researchers can gain valuable insights into optimizing patient outcomes and contributing to the ongoing discourse in restorative dentistry. The overarching purpose of this review is to consolidate existing knowledge, present current trends, and identify gaps in understanding within the realm of cast post retention. By critically analyzing historical practices, contemporary approaches, and emerging technologies, this review aims to contribute to the ongoing discourse in restorative dentistry. Additionally, it seeks to guide future research directions and inform clinical practices, ultimately enhancing the overall efficacy and longevity of cast post-retained restorations.

## Review

Factors influencing cast post retention

Tooth-Related Factors

Root canal anatomy: Root canal anatomy plays a pivotal role in determining the feasibility and success of cast post retention. The intricacies of the root canal system, including the number of canals, their curvature, and the presence of isthmuses, significantly influence the ease of post placement and the overall stability of the restoration. Clinicians must carefully assess and adapt their approach based on the unique anatomical features of each tooth [4].

Tooth type and location: Different teeth exhibit distinct characteristics, and their location within the oral cavity can impact the forces and stresses they are subjected to. Anterior teeth, for instance, may experience different functional demands than posterior teeth. The type of tooth and its location influence the selection of appropriate post materials, lengths, and diameters, as well as the overall treatment approach [5].

Presence of ferrule effect: The ferrule effect, defined as the existence of a continuous band of tooth structure encircling the coronal aspect of a tooth, has substantial implications for cast post retention. A well-established ferrule provides increased resistance to dislodgment forces and contributes to the overall stability of the restoration. A ferrule's absence or inadequate presence may compromise the success of the cast post-retained restoration, necessitating careful consideration during treatment planning [6].

Post-Related Factors

Material selection: The selection of an appropriate material for the cast post is a critical decision that significantly impacts the mechanical and biological aspects of the restoration. Common materials for cast posts include gold alloys, titanium, and base metals like stainless steel. Each material possesses unique properties, such as strength, corrosion resistance, and biocompatibility. The choice of material should be aligned with the specific requirements of the clinical case, considering factors such as aesthetics, patient preferences, and the need for radiopacity in diagnostic imaging [7]. In this context, when referring to stainless steel, it is essential to clarify that it falls under the category of base metals commonly used in dental prosthetics.

Design and geometry: The design and geometry of the cast post play a significant role in the distribution of functional and nonfunctional forces within the tooth structure. Factors such as post length, diameter, and taper contribute to the overall stability of the restoration. An understanding of biomechanics is crucial in determining the appropriate design parameters. Innovations in post-design, including tapered posts, may enhance the root fracture, aiming to enhance retention while minimizing the risk of root fracture [8].

Surface treatment: Surface treatment of the cast post is essential for promoting adhesion to the luting cement and optimizing the bond with the root dentin. Surface modifications, such as sandblasting, acid etching, or the application of adhesive primers, enhance the surface energy and create a microretentive surface for the cement. Effective surface treatment improves the bond strength between the post and the surrounding tooth structure, contributing to the overall stability and success of the restoration [9].

Cementation Techniques

Cement types and properties: The choice of cement is pivotal in achieving a durable and reliable cast post-retained restoration. Various types of cement are available, including zinc phosphate, glass ionomer, resin-modified glass ionomer, and resin cement. Each type has distinct properties related to strength, adhesion, solubility, and biocompatibility. The cement selected should align with the material of the post, the tooth structure present, and the specific clinical requirements of the case. Recent advancements in adhesive technologies have introduced self-adhesive resin cement, offering simplified application procedures without compromising bond strength [10].

Cementation protocols: Cementation protocols encompass the step-by-step procedures for applying the chosen cement and seating the cast post. This includes proper mixing and manipulation of the cement, adequate cleaning and conditioning of the post and post space, not root canal, and meticulous post seating within the canal. The use of proper isolation techniques, such as rubber dam placement, is crucial to prevent contamination during the cementation process. Adherence to standardized protocols ensures optimal cement properties and facilitates the establishment of a strong bond between the post and the surrounding tooth structure [11].

Adhesion to dentin: Achieving reliable adhesion between the cement and dentin is fundamental to the success of cast post retention. The dentin surface should be appropriately prepared to create a microretentive environment for the cement. Conditioners, such as dental adhesives or etchants, may be employed to improve the bond strength between the cement and dentin. Developing a durable bond is critical for resisting dislodgment forces and preventing microleakage, ultimately influencing the long-term stability of the post-retained restoration [12].

Prosthesis-Related Factors

Crown material and design: The material and design of the crown represent pivotal aspects of the prosthetic phase in cast post-retained restorations. The selection of crown material, such as metal-ceramic, all-ceramic, or metal-free alternatives, is influenced by aesthetic demands, functional requirements, and patient preferences. The design of the crown, including margin placement, contour, and occlusal anatomy, directly affects the stress distribution within the restoration. Understanding the interplay between the crown and the supporting cast post is crucial for achieving a harmonious and durable prosthesis [13].

Occlusion and function: The relationship between occlusion and function is critical in post-retained restorations. Occlusal forces exerted during mastication impact the load distribution within the restoration and, subsequently, on the supporting tooth and cast post. Careful assessment of occlusal dynamics, including articulation patterns, centric and eccentric contacts, and potential interferences, is essential to prevent premature wear, fracture, or dislodgment of the restoration. Occlusal adjustments and equilibration may be necessary to optimise the functional aspects of the cast post-retained prosthesis [14].

Restoration longevity: Evaluating the long-term success and durability of the restoration is paramount. Prosthesis-related factors, such as material wear, degradation, or mechanical failure, contribute to the overall longevity of the restoration. Regular follow-up examinations and maintenance protocols are crucial for identifying and addressing potential issues early in the lifespan of the cast post-retained restoration. Additionally, patient education regarding oral hygiene practices and the avoidance of deleterious habits can significantly extend the restoration of functional life [15].

Clinical techniques for cast post retention

Step-by-step Procedure for Cast Post Placement

The clinical procedure for cast post placement is a meticulous process that requires precision and adherence to established protocols. This step-by-step guide outlines the critical phases in achieving successful cast post retention. Table 1 describes the step-by-step procedure for cast post placement [16].

**Table 1:** Outlining the step-by-step procedure for cast post-placement

Step	Procedure
Preoperative Assessment	A comprehensive evaluation of tooth condition, including pulp vitality and periapical status. Radiographic assessment to determine root canal anatomy and any existing pathology
Tooth Preparation	Access cavity preparation allows for thorough root canal system cleaning and shaping. Removal of any remaining coronal structure, ensuring adequate ferrule effect
Root Canal Treatment	Cleaning and shaping the root canal system to facilitate optimal post-placement. Obturation of the canal with a suitable endodontic sealer and gutta-percha
Post Space Preparation	Create a post space corresponding to the selected post's dimensions without compromising the remaining tooth structure. Irrigation and drying of the post space to remove debris and disinfect
Post Selection	Consideration of the tooth-related factors (e.g., root canal anatomy, tooth type) and post-related factors (e.g., material, design) in selecting an appropriate post
Surface Treatment	Application of surface treatments to enhance the post's adhesion to cement, such as sandblasting or acid etching
Cementation	Selection of an appropriate cement based on the post material and clinical requirements. Careful mixing and application of the cement following established protocols. Precise seating of the post within the prepared post space
Crown Preparation	Tooth reduction and preparation for the final restoration, considering aesthetics and occlusal factors
Impression and Temporization	Impressions for the fabrication of the final restoration. Provisional restoration to protect the prepared tooth until the final prosthesis is ready
Final Restoration Placement	Cementation of the final restoration onto the cast post. Occlusal adjustments to ensure proper function and comfort

Common Challenges and Solutions

Despite carefully executing the procedure, clinicians may encounter challenges in cast post retention. Addressing these challenges promptly is essential for a successful outcome. Common challenges and their solutions are described in Table 2 [17].

**Table 2:** Common challenges and their solutions

Complication	Solution
Fracture of the Remaining Tooth Structure	Reinforce the remaining tooth structure with additional restorative materials, such as composite resin or amalgam. Alternatively, consider alternative approaches like a core buildup to provide additional support and prevent further fracture.
Inadequate Ferrule Effect	Evaluate the extent of the remaining tooth structure and, if possible, modify the preparation to enhance the ferrule effect. Extending the crown margin or adjusting the tooth preparation can improve the retention and stability of the cast post-retained restoration. Ensuring an adequate ferrule is crucial for the long-term success of the restoration.
Post Dislodgment during Cementation	Ensure the proper fit of the post within the post space. Use a high-quality cement compatible with the post material and the tooth structure. Consider post-surface treatments, such as sandblasting or acid etching, to improve the adhesion between the post and the cement. Additionally, verify that the cementation protocol is followed meticulously to secure the post.
Post Space Complications	Address any difficulties encountered during post-space preparation. Use rotary instruments appropriate for the chosen post material to ensure precision and prevent complications. Adjust the technique or instruments to create a well-prepared post space that accommodates the selected post without compromising the remaining tooth structure.

Innovations in Cast Post-retention Techniques

Continual advancements in dental technology and materials have led to innovations in cast post-retention techniques. These innovations aim to address challenges, improve outcomes, and enhance the overall efficiency of the procedure. Notable innovations are described in Table 3 [18].

**Table 3:** Advancements in technology for cast post retention

Technology	Description
Digital Impression Techniques	Implementing intraoral scanners for accurate and efficient impressions reduces the need for traditional impression materials. Digital impressions provide a highly accurate virtual model of the oral environment, enhancing precision and comfort for clinicians and patients.
CAD/CAM Technology	Utilisation of computer-aided design and manufacturing (CAD/CAM) for the fabrication of custom cast posts. CAD/CAM technology ensures precision and consistency in post design, allowing for the creation of highly customised posts that match the patient's unique anatomical features. This technology streamlines the workflow and enhances the overall efficiency of cast post-retention procedures.
Biocompatible and Aesthetic Materials	Development of new materials, such as zirconia posts, that offer improved aesthetics and biocompatibility while maintaining strength. These advanced materials provide clinicians with versatile options for post fabrication, addressing both functional and aesthetic considerations in restorative dentistry.
3D Printing in Post Fabrication	Integration of three-dimensional (3D) printing technologies to create custom-designed posts based on digital models. 3D printing allows for the fabrication of patient-specific solutions, accommodating the unique anatomical features of each tooth. This technology enhances precision and opens innovative design possibilities in cast post retention.

Clinical outcomes and long-term studies

Success Rates of Cast Post-Retained Restorations

Several studies have been conducted to evaluate the success rates of cast post-retained restorations. A retrospective study on 140 teeth aimed to determine the survival and success rates of endodontically treated teeth restored with cast post and core. The study found that the success rate was around one-third of the total sample, while the survival rate exceeded two-thirds [19]. Another study evaluated the success rates of post and core-treated anterior and posterior teeth using cast metal posts. The study found that the success rate of post and core-treated anterior teeth was higher than that of posterior teeth [20].

However, other studies have reported varying success rates ranging from 55% to 72.9% [21-23]. Factors such as post diameter, post length, stress distribution, and post design play a significant role in the retention of metal posts [22]. Additionally, biomechanical failures such as post-loosening and tooth fractures are the most frequent failures [21]. The success rates of cast post-retained restorations vary depending on several factors, and long-term follow-up studies have provided valuable insights into the longevity and risk factors associated with post restorations. Understanding these factors and long-term outcomes is essential for clinicians to make informed decisions and achieve successful long-term prognoses for post-and-core-retained restorations.

Complications and Failures

Recurrent caries and apical periodontitis are less prevalent [21]. Long-term follow-up studies have shown varying success rates, ranging from 55% to 90.6% after six years [24]. A 44-year retrospective clinical study in a specialized private clinic found that the cumulative survival rate of cast metal core restorations was 80.5% [21]. The complications and failures associated with cast post-retained restorations include biomechanical failures such as post-loosening and tooth fractures, the most frequent failures. Long-term follow-up studies have shown varying success rates, and understanding these factors is crucial for achieving long-term prognosis for post-and-core retained restorations.

Long-term Follow-up Studies

Long-term follow-up studies have provided valuable insights into the longevity and risk factors associated with post-restorations. A practice-based study examined the longevity and risk factors of post-restorations after up to 15 years, shedding light on the long-term clinical outcomes of endodontically treated teeth restored with or without fibre post-retained single-unit restorations [23-24]. Additionally, a clinical study with up to nine years of follow-up compared glass fibre post- and cast-metal post-retained restorations, providing further insights into the long-term survival of adhesively luted post-endodontic restorations [25]. Furthermore, a 44-year retrospective clinical study in a specialized private clinic found that the cumulative survival rate of cast metal core restorations was 80.5% [26]. These studies contribute to understanding the long-term clinical outcomes and survival rates of post-retained restorations, providing valuable information for clinicians in making informed decisions regarding post-and-core retained restorations. The long-term follow-up studies have provided valuable insights into the longevity and risk factors associated with post-restorations, contributing to understanding long-term clinical outcomes and survival rates of post-retained restorations.

Advancements in technology

Digital Impressions and Computer-Aided Design and Computer-Aided Manufacturing (CAD/CAM) Technology

Precision and accuracy: Adopting digital impressions marks a revolutionary leap in achieving unparalleled precision and accuracy in cast post-retention procedures. Traditional impression materials are prone to distortion, often compromising the accuracy of the obtained models. Digital impressions eradicate this limitation, accurately representing the tooth and its surrounding structures. The virtual models generated through digital impressions are an intricate blueprint, laying the foundation for meticulous treatment planning and execution in cast post retention. This heightened precision ensures that the cast post aligns seamlessly with the patient's unique anatomical features, contributing to the overall success and longevity of the restoration [27].

Time efficiency: Integrating intraoral scanners and CAD/CAM technology significantly boosts time efficiency for casting post-retention procedures. Unlike traditional impression-taking methods that involve cumbersome materials and extended waiting times, the digital workflow streamlines the entire process. Intraoral scanners swiftly capture detailed impressions, and CAD/CAM technology expedites subsequent model fabrication. This enhances the overall efficiency of cast post-retention procedures and translates to a more time-conscious and convenient experience for clinicians and patients. Reducing chair time improves patient comfort and satisfaction while allowing clinicians to optimize their workflow [28].

Customization: CAD/CAM technology emerges as a critical enabler in customising post design based on the digital impressions acquired. This level of customization empowers clinicians to tailor the cast post to the specific anatomical features of the tooth, ensuring an optimal fit and alignment. The digital information obtained through intraoral scanners facilitates a detailed analysis of the tooth's structure, enabling precise adjustments to meet individual patient requirements. The ability to customise post design based on this precise digital information significantly contributes to the overall success and longevity of the restoration, fostering a more personalized and effective treatment approach [29].

Provisional restorations: Digital impressions facilitate the efficient creation of provisional restorations, offering immediate protection to the prepared tooth while the final restoration is in progress. This capability is particularly beneficial in maintaining the integrity of the tooth structure during the restoration process. By swiftly generating digital impressions, clinicians can promptly craft provisional restorations that align with patients' needs. This not only enhances the overall patient experience by providing interim solutions but also contributes to the success of the final restoration. The capability to deliver provisional restorations efficiently represents a significant advancement in patient care and treatment continuity in cast post-retention procedures [30].

Three-dimensional (3D) Printing in Post Fabrication

Patient-specific design: One of the hallmark advantages of integrating 3D printing into cast post fabrication is its capacity to facilitate patient-specific design. Through digital models, 3D printing enables clinicians to embark on a tailored approach, crafting cast posts that are meticulously designed to accommodate the unique anatomical features of each tooth. Cast post is substantially more accurate than 3D printing as the scanner may not get the details of post space in deep spaces. As a result, the restoration process becomes inherently more personalized, enhancing not only the fit and function of the cast post but also contributing to the overall precision and success of the restorative procedure [31].

Material variety: Three-dimensional printing introduces a paradigm of material variety, offering clinicians a diverse selection of biocompatible materials for cast post fabrication. This versatility allows practitioners to choose materials based on specific clinical requirements, tailoring the cast post to meet distinct needs. Whether prioritizing factors such as strength, biocompatibility, or aesthetic considerations, the array of materials available in 3D printing empowers clinicians to adopt a nuanced and optimized approach. The flexibility to select materials best suited to individual cases enhances the overall quality and functionality of the cast post-retained restoration, addressing diverse patient needs with a customised and precise solution [32].

Rapid prototyping: The revolutionary aspect of 3D printing in cast post fabrication lies in its rapid prototyping capabilities, fundamentally altering traditional production timelines. This accelerated process diminishes the time typically associated with cast post fabrication, allowing for the swift delivery of custom-designed cast posts to the clinical setting. Reducing production time enhances workflow efficiency for clinicians and ensures a more expeditious restoration process for patients. This timelier approach improves overall treatment experiences, reducing patient waiting periods and facilitating a quicker transition from the preparatory phase to the final restoration, enhancing patient satisfaction and streamlining clinical workflows [33].

Complex geometries: Three-dimensional printing excels in accommodating complex geometries, presenting a significant advantage in adapting the cast post to the intricate root canal anatomy. The precision of 3D printing technology enables the fabrication of highly detailed and complex structures, ensuring that the cast post conforms seamlessly to the unique contours of the tooth. This capability is particularly beneficial in cases where traditional fabrication methods may need help to capture the nuances of intricate root canal systems. By embracing complex geometries, 3D printing contributes to the biomechanical efficacy and overall success of the cast post-retained restoration, offering a level of adaptability and precision that enhances the long-term stability and functionality of the restoration [34].

Computer-Aided Finite Element Analysis in Post Design

Biomechanical optimization: One of the primary advantages of computer-aided engineering and finite element analysis (CAE/FEA) is its capability to conduct a comprehensive analysis of stress points and load distribution within the restoration. By simulating the mechanical forces acting on the cast post-retained restoration, clinicians understand how stresses are distributed throughout the tooth and surrounding structures. This insight aids in optimizing post design, allowing for adjustments that enhance the biomechanical performance of the restoration. CAE/FEA provides precision that goes beyond traditional approaches, contributing to the overall stability and functionality of the cast post-retained restoration [35].

Material selection guidance: CAE/FEA offers valuable guidance in selecting materials for both the post and the restoration. By simulating material interactions, clinicians can assess how different materials behave under various loading conditions. This information assists in making informed decisions about material selection, considering factors such as strength, compatibility, and the overall performance of the materials within the specific context of the cast post-retained restoration. The ability to optimize material combinations contributes to the longevity and success of the restoration [36].

Risk assessment: The simulation capabilities of CAE/FEA enable clinicians to identify potential weak points or areas of high-stress concentration within the restoration. By conducting risk assessments through virtual scenarios, clinicians can proactively identify and address issues that may compromise the structural integrity of the cast post-retained restoration. This preemptive risk assessment allows for adjustments in the post design, mitigating potential challenges before they manifest clinically. CAE/FEA is a robust risk management tool, contributing to the overall reliability of the restoration [37].

Predictive modelling: Clinicians can leverage CAE/FEA to create predictive models of the long-term performance of the cast post-retained restoration under different loading conditions. By simulating the effects of various forces over time, clinicians gain insights into how the restoration is likely to behave in the clinical setting. This evidence-based approach to predictive modelling enhances treatment planning, allowing clinicians to anticipate the behaviour of the restoration and make informed decisions about post design and material selection. Predictive modelling contributes to establishing realistic expectations for the longevity and success of the cast post-retained restoration [38].

Future directions

Emerging Materials and Techniques

Advanced biomaterials: The forefront of research involves the exploration of novel biomaterials designed to mimic the natural tooth structure and foster better integration with surrounding tissues. These advanced biomaterials aim to provide a more harmonious interface between the cast post and the tooth, potentially reducing the risk of complications and enhancing the overall success of restorations. The pursuit of biomimicry holds promise in creating materials that function effectively and contribute to the restored tooth's overall health and stability [39].

Nanostructured materials: Investigations into nanostructured materials mark a significant stride towards improving cast posts' mechanical properties and bioactivity. By leveraging nanotechnology, researchers aim to enhance the structural integrity of cast posts, potentially leading to superior long-term performance. The nanostructured approach holds the potential to optimize material strength, reduce susceptibility to wear, and promote better interaction with the surrounding biological environment, fostering a more durable and biocompatible restoration [40].

Biodegradable materials: Exploring biodegradable materials introduces a paradigm shift in cast post-retention strategies. Research in this domain focuses on materials that can gradually resorb, providing temporary support structures before natural tissue integration occurs. This approach aligns with the principles of tissue regeneration. It may offer advantages in minimizing long-term complications, reducing the need for secondary interventions and promoting a more natural integration with the surrounding dental structures [41].

Hybrid composites: The development of hybrid composite materials represents a convergence of benefits, such as improved strength and aesthetics. By combining the advantageous properties of diverse materials, researchers seek to create hybrid composites that offer a synergistic blend of strength, durability, and aesthetic appeal. This innovative approach addresses the multifaceted requirements of cast post retention, providing clinicians with versatile and high-performance materials for restorative applications [42].

Integration of Artificial Intelligence (AI) in Treatment Planning

Diagnostic imaging and analysis: AI algorithms have demonstrated exceptional capabilities in enhancing the analysis of diagnostic imaging. In the context of cast post retention, this entails a more refined assessment of root canal anatomy and precise identification of optimal post-placement sites. By leveraging AI, clinicians can benefit from a more nuanced understanding of the intricacies of each patient's tooth structure, facilitating strategic decision-making in placing cast posts [43].

Treatment simulation: The application of AI-powered simulations is a valuable tool for predicting the biomechanical behaviour of different post designs. This innovation allows clinicians to virtually simulate the performance of various cast post options, aiding in selecting the most suitable design for specific cases. By providing insights into how different designs interact with the unique characteristics of each tooth, AI-driven simulations empower clinicians to make informed decisions, enhancing the overall biomechanical success of cast post-retained restorations [44].

Customised treatment plans: One of the noteworthy contributions of AI lies in its ability to assist in developing customised treatment plans tailored to patient-specific factors. AI can optimize post-selection by analyzing many variables, including patient demographics, oral health history, and anatomical considerations. This personalized approach ensures that the chosen cast post aligns seamlessly with the patient's characteristics, contributing to a more tailored and effective treatment plan [45].

Outcome prediction: AI models extend their utility by offering predictions regarding the long-term success of cast post-retained restorations. These models guide clinicians in treatment planning and patient counselling by considering patient demographics, material properties, and historical treatment outcomes. The ability to predict outcomes allows for a more comprehensive and proactive approach to post-retained restorations, potentially reducing the likelihood of complications and improving overall treatment predictability [46-47].

Patient-Specific Approaches to Cast Post Retention

Behavioural analysis: Integrating behavioural analysis represents a holistic approach to cast post retention, extending beyond anatomical considerations. By assessing oral hygiene practices, compliance, and potential risk factors for restoration failure, clinicians gain valuable insights into the behavioural aspects that can impact the success of cast post-retained restorations. This patient-centric analysis allows targeted interventions and personalized guidance to improve long-term outcomes [48].

Virtual treatment planning: The development of virtual treatment planning platforms marks a technological leap in ensuring patient-specific anatomical variations and preferences are considered. These platforms enable clinicians to create virtual models that simulate the patient's unique oral environment. By incorporating anatomical nuances and individual preferences, virtual treatment planning ensures a more personalized and precise approach to cast post retention, improving treatment efficacy [49].

Patient education and involvement: Empowering patients through education and active involvement in decision-making fosters a collaborative approach to treatment planning. Clinicians can enhance patient compliance and cooperation by giving patients a deeper understanding of the cast post-retention process, including potential risks and benefits. This collaborative engagement ensures informed decision-making and contributes to long-term success by cultivating a sense of shared responsibility between the clinician and the patient [50].

## Conclusions

In conclusion, this comprehensive review has thoroughly explored the multifaceted considerations surrounding cast post retention in restorative dentistry. From historical perspectives to contemporary techniques, the analysis of tooth-related factors, post-related factors, cementation techniques, and prosthesis-related factors has illuminated the complexities involved in achieving successful outcomes. Examining clinical techniques, including step-by-step procedures, common challenges, and innovations, has offered valuable insights into the practical aspects of cast post placement. Furthermore, the discussion on technological advancements, such as digital impressions, CAD/CAM technology, 3D printing, and finite element analysis, has highlighted the transformative potential of these tools in optimizing precision and customization. Looking ahead, the future of cast post retention holds promise with the exploration of emerging materials, integration of AI in treatment planning, and patient-specific approaches. The implications for clinical practice underscore the importance of individualized treatment planning and the incorporation of advanced technologies. Recommendations for future research emphasize the need for long-term clinical studies, AI-driven treatment planning investigations, and a focus on patient outcomes and satisfaction. In essence, this review consolidates existing knowledge and points towards an exciting future characterized by enhanced precision, individualized care, and improved long-term outcomes for patients undergoing cast post-retained restorations.

## References

[1] Carvalho MA, Lazari PC, Gresnigt M, Del Bel Cury AA, Magne P (2018). Current options concerning the endodontically-treated teeth restoration with the adhesive approach. Braz Oral Res.

[2] Al-Qarni FD (2022). Customized post and cores fabricated with CAD/CAM technology: a literature review. Int J Gen Med.

[3] Spielman H, Schaffer SB, Cohen MG (2012). Restorative outcomes for endodontically treated teeth in the Practitioners Engaged in Applied Research and Learning network. J Am Dent Assoc.

[4] Al Yahya RS, Al Attas MH, Javed MQ, Khan KI, Atique S, Abulhamael AM, Bahammam HA (2023). Root canal configuration and its relationship with endodontic technical errors and periapical status in premolar teeth of a Saudi sub-population: a cross-sectional observational CBCT study. Int J Environ Res Public Health.

[5] Lacruz RS, Habelitz S, Wright JT, Paine ML (2017). Dental enamel formation and implications for oral health and disease. Physiol Rev.

[6] Juloski J, Radovic I, Goracci C, Vulicevic ZR, Ferrari M (2012). Ferrule effect: a literature review. J Endod.

[7] Saini M, Singh Y, Arora P, Arora V, Jain K (2015). Implant biomaterials: a comprehensive review. World J Clin Cases.

[8] Ausiello P, Gloria A, Maietta S, Watts DC, Martorelli M (2020). Stress distributions for hybrid composite endodontic post designs with and without a ferrule: FEA study. Polymers (Basel).

[9] D'Arcangelo C, Vanini L (2007). Effect of three surface treatments on the adhesive properties of indirect composite restorations. J Adhes Dent.

[10] Ghodsi S, Aghamohseni MM, Arzani S, Rasaeipour S, Shekarian M (2022). Cement selection criteria for different types of intracanal posts. Dent Res J (Isfahan).

[11] Hoshino IA, Gallinari MO, Mestrener SR, Anchieta NR, Anchieta RB (2020). An alternative 2-step cementation technique to reduce polymerization shrinkage stress in root canals: a case report. Gen Dent.

[12] Perdigão J (2020). Current perspectives on dental adhesion: (1) dentin adhesion - not there yet. Jpn Dent Sci Rev.

[13] Zhang Y, Kelly JR (2017). Dental ceramics for restoration and metal-veneering. Dent Clin North Am.

[14] D’Amico C, Bocchieri S, Sambataro S, Surace G, Stumpo C, Fiorillo L (2020). Occlusal load considerations in implant-supported fixed restorations. Prosthesis.

[15] Demarco FF, Corrêa MB, Cenci MS, Moraes RR, Opdam NJ (2012). Longevity of posterior composite restorations: not only a matter of materials. Dent Mater.

[16] Ghidrai G (2023). Ghidrai G: How is a Cast Post and Core Manufactured?. https://www.infodentis.com/post-and-core/cast-post-and-core-procedure.php.

[17] (2004). The necessity and challenges of clinical research involving children. Ethical Conduct of Clinical Research Involving Children.

[18] Prithviraj DR, Bhalla HK, Vashisht R, Sounderraj K, Prithvi S (2014). Revolutionizing restorative dentistry: an overview. J Indian Prosthodont Soc.

[19] Subait AA, Albawardi A, Alghomlas A, Daabash M, Alotaibi M, Alturki Y (2016). Success and survival rates of teeth restored with cast post and core among national guard health affairs patients, Riyadh, Saudi Arabia. Adv Dent Oral Health.

[20] Prakash J, Golgeri MS, Haleem S, Kausher H, Gupta P, Singh P, C SG (2022). A comparative study of success rates of post and core treated anterior and posterior teeth using cast metal posts. Cureus.

[21] Botto EB, Barón R, Borgia JL (2015). Cast posts: a 44-year retrospective clinical study in a specialized private clinic. SciELO Uruguay.

[22] Peutzfeldt A, Sahafi A, Asmussen E (2008). A survey of failed post-retained restorations. Clin Oral Investig.

[23] Linnemann T, Kramer EJ, Schwendicke F, Wolf TG, Meyer-Lueckel H, Wierichs RJ (2021). Longevity and risk factors of post restorations after up to 15 years: a practice-based study. J Endod.

[24] de Andrade GS, Saavedra GD, Augusto MG, Leon GA, Brandão HC, Tribst JP, Piva AM (2023). Post-endodontic restorative treatments and their mechanical behavior: a narrative review. Dent Rev.

[25] Bruhnke M, Wierichs RJ, von Stein-Lausnitz M, Meyer-Lückel H, Beuer F, Naumann M, Sterzenbach G (2022). Long-term survival of adhesively luted post-endodontic restorations. J Endod.

[26] Fokkinga WA, Kreulen CM, Bronkhorst EM, Creugers NH (2007). Up to 17-year controlled clinical study on post-and-cores and covering crowns. J Dent.

[27] Pesce P, Pera F, Setti P, Menini M (2018). Precision and accuracy of a digital impression scanner in full-arch implant rehabilitation. Int J Prosthodont.

[28] Patzelt SB, Lamprinos C, Stampf S, Att W (2014). The time efficiency of intraoral scanners: an in vitro comparative study. J Am Dent Assoc.

[29] Berrendero S, Salido MP, Valverde A, Ferreiroa A, Pradíes G (2016). Influence of conventional and digital intraoral impressions on the fit of CAD/CAM-fabricated all-ceramic crowns. Clin Oral Investig.

[30] Regish KM, Sharma D, Prithviraj DR (2011). Techniques of fabrication of provisional restoration: an overview. Int J Dent.

[31] Aimar A, Palermo A, Innocenti B (2019). The role of 3D printing in medical applications: a state of the art. J Healthc Eng.

[32] Pillai S, Upadhyay A, Khayambashi P (2021). Dental 3D-printing: transferring art from the laboratories to the clinics. Polymers (Basel).

[33] Ngo TD, Kashani A, Imbalzano G, Nguyen KT, Hui D (2018). Additive manufacturing (3D printing): a review of materials, methods, applications and challenges. Compos Part B Eng.

[34] Reis T, Barbosa C, Franco M (2022). 3D-printed teeth in endodontics: why, how, problems and future-a narrative review. Int J Environ Res Public Health.

[35] Trivedi S (2014). Finite element analysis: a boon to dentistry. J Oral Biol Craniofac Res.

[36] Alaneme KK, Kareem SA, Ozah BN, Alshahrani HA, Ajibuwa OA (2022). Application of finite element analysis for optimizing selection and design of Ti-based biometallic alloys for fractures and tissues rehabilitation: a review. J Mater Res Technol.

[37] Khadar S, Sapkale K, Patil PG, Abrar S, Ramugade M, Huda F (2022). Fracture resistance and stress distribution pattern of different posts-core systems in immature teeth: an in vitro study and 3D finite element analysis. Int J Dent.

[38] Sorrentino R, Salameh Z, Zarone F, Tay FR, Ferrari M (2007). Effect of post-retained composite restoration of MOD preparations on the fracture resistance of endodontically treated teeth. J Adhes Dent.

[39] Matichescu A, Ardelean LC, Rusu LC, Craciun D, Bratu EA, Babucea M, Leretter M (2020). Advanced biomaterials and techniques for oral tissue engineering and regeneration-a review. Materials (Basel).

[40] Jeevanandam J, Barhoum A, Chan YS, Dufresne A, Danquah MK (2018). Review on nanoparticles and nanostructured materials: history, sources, toxicity and regulations. Beilstein J Nanotechnol.

[41] Llorens E, Armelin E, Pérez-Madrigal MD, Valle LJ, Alemán C, Puiggalí J (2013). Nanomembranes and nanofibers from biodegradable conducting polymers. Polymers.

[42] Zhang J, Chaisombat K, He S, Wang CH (198020152012). Hybrid composite laminates reinforced with glass/carbon woven fabrics for lightweight load bearing structures. Mater Des.

[43] Tang X (2020). The role of artificial intelligence in medical imaging research. BJR Open.

[44] Evans LM, Sözümert E, Keenan BE, Wood CE, du Plessis A (2023). A review of image-based simulation applications in high-value manufacturing. Arch Comput Methods Eng.

[45] Nosrati H, Nosrati M (2023). Artificial intelligence in regenerative medicine: applications and implications. Biomimetics (Basel).

[46] Karobari MI, Adil AH, Basheer SN (2023). Evaluation of the diagnostic and prognostic accuracy of artificial intelligence in endodontic dentistry: a comprehensive review of literature. Comput Math Methods Med.

[47] Tulek A, Mulic A, Runningen M, Lillemo J, Utheim TP, Khan Q, Sehic A (2021). Genetic aspects of dental erosive wear and dental caries. Int J Dent.

[48] Adair PM, Burnside G, Pine CM (2013). Analysis of health behaviour change interventions for preventing dental caries delivered in primary schools. Caries Res.

[49] Swennen GR, Mollemans W, Schutyser F (2009). Three-dimensional treatment planning of orthognathic surgery in the era of virtual imaging. J Oral Maxillofac Surg.

[50] Chen J, Mullins CD, Novak P, Thomas SB (2016). Personalized strategies to activate and empower patients in health care and reduce health disparities. Health Educ Behav.

